# Ex vivo piperaquine resistance developed rapidly in *Plasmodium falciparum* isolates in northern Cambodia compared to Thailand

**DOI:** 10.1186/s12936-016-1569-y

**Published:** 2016-10-21

**Authors:** Suwanna Chaorattanakawee, Chanthap Lon, Krisada Jongsakul, Jariyanart Gawee, Somethy Sok, Siratchana Sundrakes, Nareth Kong, Chatchadaporn Thamnurak, Soklyda Chann, Sorayut Chattrakarn, Chantida Praditpol, Nillawan Buathong, Nichapat Uthaimongkol, Philip Smith, Narongrid Sirisopana, Rekol Huy, Satharath Prom, Mark M. Fukuda, Delia Bethell, Douglas S. Walsh, Charlotte Lanteri, David Saunders

**Affiliations:** 1 US Army Medical Component-Armed Forces Research Institute of Medical Sciences (USAMC-AFRIMS), Bangkok, Thailand; 2 Department of Parasitology and Entomology, Faculty of Public Health, Mahidol University, Bangkok, Thailand; 3 USAMC-AFRIMS, Phnom Penh, Cambodia; 4 Royal Thai Army, Bangkok, Thailand; 5 Royal Cambodian Armed Forces, Phnom Penh, Cambodia; 6 National Center for Parasitology, Entomology and Malaria Control, Phnom Penh, Cambodia; 7 Department of Pathology and Area Laboratory Services, Microbiology Section, Brooke Army Medical Center, San Antonio, TX USA

**Keywords:** Malaria, Drug resistance, Piperaquine, Mefloquine, Cambodia, Thailand

## Abstract

**Background:**

The recent dramatic decline in dihydroartemisinin-piperaquine (DHA-PPQ) efficacy in northwestern Cambodia has raised concerns about the rapid spread of piperaquine resistance just as DHA-PPQ is being introduced as first-line therapy in neighbouring countries.

**Methods:**

Ex vivo parasite susceptibilities were tracked to determine the rate of progression of DHA, PPQ and mefloquine (MQ) resistance from sentinel sites on the Thai–Cambodian and Thai–Myanmar borders from 2010 to 2015. Immediate ex vivo (IEV) histidine-rich protein 2 (HRP-2) assays were used on fresh patient *Plasmodium falciparum* isolates to determine drug susceptibility profiles.

**Results:**

IEV HRP-2 assays detected the precipitous emergence of PPQ resistance in Cambodia beginning in 2013 when 40 % of isolates had an IC_90_ greater than the upper limit of prior years, and this rate doubled to 80 % by 2015. In contrast, Thai–Myanmar isolates from 2013 to 14 remained PPQ-sensitive, while northeastern Thai isolates appeared to have an intermediate resistance profile. The opposite trend was observed for MQ where Cambodian isolates appeared to have a modest increase in overall sensitivity during the same period, with IC_50_ declining to median levels comparable to those found in Thailand. A significant association between increased PPQ IC_50_ and IC_90_ among Cambodian isolates with DHA-PPQ treatment failure was observed. Nearly all Cambodian and Thai isolates were deemed artemisinin resistant with a >1 % survival rate for DHA in the ring-stage assay (RSA), though there was no correlation among isolates to indicate cross-resistance between PPQ and artemisinins.

**Conclusions:**

Clinical DHA-PPQ failures appear to be associated with declines in the long-acting partner drug PPQ, though sensitivity appears to remain largely intact for now in western Thailand. Rapid progression of PPQ resistance associated with DHA-PPQ treatment failures in northern Cambodia limits drugs of choice in this region, and urgently requires alternative therapy. The temporary re-introduction of artesunate AS-MQ is the current response to PPQ resistance in this area, due to inverse MQ and PPQ resistance patterns. This will require careful monitoring for re-emergence of MQ resistance, and possible simultaneous resistance to all three drugs (AS, MQ and PPQ).

**Electronic supplementary material:**

The online version of this article (doi:10.1186/s12936-016-1569-y) contains supplementary material, which is available to authorized users.

## Background

Artemisinin combination therapy (ACT) remains the most effective currently available regimen for multi-drug-resistant falciparum malaria, and as such is key to reducing the global malaria burden [1, 2]. While artemisinin-resistant *Plasmodium falciparum* has emerged in the western provinces of Cambodia, and some neighboring countries in Southeast Asia [3–6], ACT appears to remain clinically effective where partner drug efficacy is preserved. Dihydroartemisinin-piperaquine (DHA-PPQ) has been widely adopted in the region as a first-line agent to treat multi-drug resistant falciparum malaria. In 2008, it replaced artesunate-mefloquine (AS-MQ) in select areas of western Cambodia due to AS-MQ treatment failures [7]. In early studies, various PPQ-containing ACT showed excellent safety and tolerability with efficacy of 96–98 % in Cambodia [8–12], and DHA-PPQ was implemented as the drug of choice countrywide a few years later [13].

Treatment failures were detected soon after, as early as 2010, with PCR-corrected day-42 failure rate of 25 % in Pailin and 11 % in Pursat Provinces in Cambodia [14]. In 2013, high grade DHA-PPQ failure of 53 % was first reported in Oddar MeanChey province with corresponding increases in PPQ in vitro IC_50_ higher than patient plasma PPQ levels during the terminal elimination phase [15–17]. Subsequent reports confirmed rapidly increasing DHA-PPQ failure rates in areas with previously documented artemisinin (ART) resistance [18, 19]. Despite the rapid loss of PPQ sensitivity, multiple studies detected a simultaneous decline in *pfmdr1* copy number amplification and increased in vitro MQ sensitivity among PPQ-resistant isolates [15, 18, 20]. The resurgence in MQ sensitivity suggested that short-term re-introduction MQ-containing therapy or perhaps triple artemisinin combination therapy (TACT) containing both PPQ and MQ might be effective. While approaches are currently being pursued to various degrees by public health authorities and research organizations [18], there is no consensus regarding safety and resistance concerns related to the use of TACT [21].

To address this growing public health crisis, intensive monitoring of drug resistance profiles remains crucial information to determine appropriate alternatives in settings of rapidly emerging multi-drug anti-malarial resistance. In the absence of a known molecular PPQ resistance marker, field-based ex vivo parasite drug susceptibility testing using fresh *P. falciparum* isolates remains a cost-effective, rapid, surveillance tool to track resistance [22]. Here, parasite drug susceptibilities to ART, PPQ and MQ between 2010 and 2015 on both sides of the Thai–Cambodian border, and in southwestern Thailand along the border with Myanmar were reported.

## Methods

### Study sites, protocols, and subjects

**Fig. 1:**
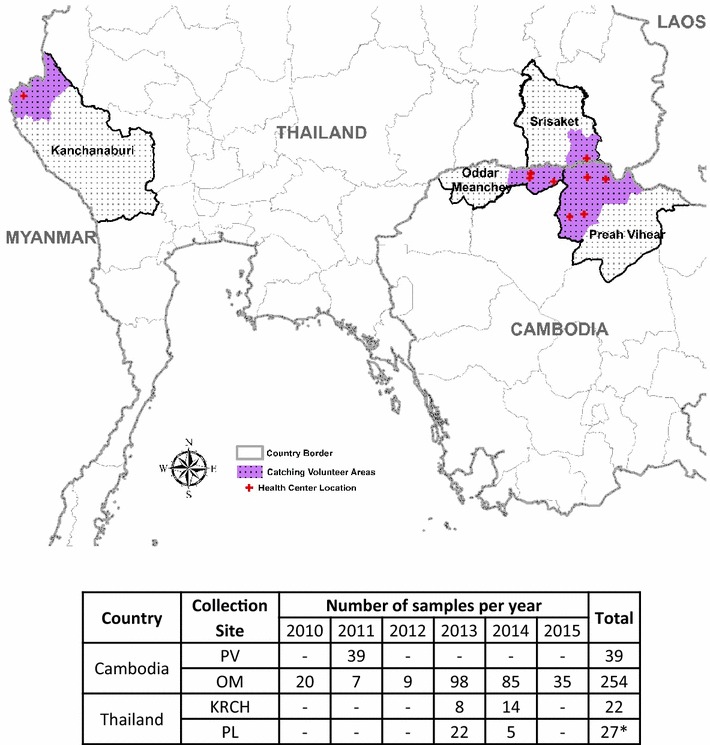
Sentinel sites for *Plasmodium falciparum* isolate collection 2010–2015. The *map* shows location of isolate collection sites: Preah Vihear (PV) and Oddar MeanChey (OM) provinces in Cambodia, the Kwai River Christian Hospital (KRCH) in Kanchanaburi province, and Pursaron (PL) village in Srisaket province, Thailand. *Dot patterns* denote collection provinces; *colour shading* indicates regional district areas, respectively, with individual health centres indicated by a plus sign. The *Table* includes collected isolates per site. *Asterisk* all samples from PV, OM and KRCH were tested fresh using the IEV assay. The 27 isolates collected from PL were initially cryopreserved; only 14 could be recovered from cryopreservation and maintained in culture to be tested for in vitro drug susceptibility

Between October 2010-April 2015, 342 *P. falciparum* mono-infections from Preah Vihear (PV) and Oddar MeanChey (OM) provinces in northern Cambodia, Pursaron (PL) village in Srisaket province in northeast Thailand, and Kwai River Christian Hospital (KRCH) in Kanchanaburi province in western Thailand were collected for evaluation for PPQ, MQ and ART susceptibility. All samples were collected from patients with uncomplicated malaria enrolled in five clinical protocols (clinical trials WR1737-NCT01280162; WR1877-NCT01849640; WR2017-NCT02052323; WR1576 and WR1917, both in vitro surveillance studies). Figure 1 shows site locations and the number of samples collected in each site per year. All protocols were approved by the Walter Reed Army Institute of Research (WRAIR) Institutional Review Board, and Cambodian National Ethics Committee for Health Research (NECHR), Institute for Development of Human Research Protection (IHRP), Ministry of Public Health, Thailand or Royal Thai Army Institutional Review Board (RTA IRB), respectively. All study subjects provided informed consent prior to participation.

### Parasite susceptibility assay to PPQ and MQ

At the PV, OM and KRCH sites, blood samples were collected prior to treatment and tested within 2–6 h for ex vivo susceptibility to PPQ and MQ using histidine rich protein-2 (HRP-2) ELISA to measure 50 and 90 % inhibitory concentration (IC_50_ and IC_90_) following previously published methods [15]. Briefly, samples with a parasitaemia of ≤0.5 % were adjusted to 1.5 % haematocrit in 0.5 % Albumax RPMI 1640 (containing 25 mM HEPES, 25 mM sodium bicarbonate, and 0.1 mg/mL gentamycin), whereas those with >0.5 % parasitaemia were diluted to the parasitaemia range of 0.2–0.5 % by adding 50 % haematocrit human O+ red blood cells in 10 % serum-RPMI 1640 and adjusted to a 1.5 % haematocrit in 0.5 % Albumax RPMI 1640 prior to adding to dried drug-coated plates. Parasites were then incubated for 72 h at 37 °C in a candle jar, after which plates were frozen and later thawed for analysis of growth inhibition using the HRP-2 ELISA. Due to limited amount of patient blood, ex vivo assay was performed as a singlet experiment. At the PL site, patient blood samples were cryopreserved in a glycerol mixed solution, then shipped to the AFRIMS laboratory in Bangkok to establish in vitro culture, and then tested for drug susceptibility as described previously [22]. Briefly, synchronized cultures with ≥90 % ring forms were diluted to 0.5 % parasitaemia with 1.5 % haematocrit in 0.5 % Albumax RPMI 1640, and transferred to dried drug-coated plates. Plates were incubated at 37 °C with 5 % CO_2_, 5 % O_2_ and 90 % N_2_ for 72 h. Assay was done for 3–5 replicates experiments and averaged values were reported. Parasite growth inhibition was assessed using the HRP-2 ELISA. As an assay benchmark, the culture of reference *P. falciparum* W2 were tested for in vitro drug susceptibility as described above. All test drugs were provided by the WRAIR (Silver Spring, MD, USA). MQ and PPQ were dissolved in 70 % ethanol and 0.5 % lactic acid in distilled water to make 1 mg/mL stock solutions, respectively. Details for preparing drug coated plates was described previously [23].

Parasite growth after 72 h was assessed by HRP-2 ELISA. HRP-2 optical density (OD) readings were plotted against drug concentrations, IC_50_s and IC_90_s were estimated by non-linear regression analysis using the Graph-Pad Prism version 6.0 (GraphPad Software, Inc.-, San Diego, CA, USA). PPQ and MQ concentrations in the assay ranged from 0.9 to 674 nM and 0.3 to 200 nM, respectively, as well as a drug-free control to assess normal parasite growth. Starting in 2013, there were evidences that field isolates were able to grow in extremely high PPQ concentrations, requiring maximum concentrations as high as 53,905 nM in order to obtain interpretable drug susceptibility curves. This was far higher than the standard maximum PPQ level used (674 nM). Therefore, since 2014, a serial dilution range to achieve higher maximum PPQ concentrations (3.4–53,905 nM) was used in addition to the standard dilutions to ensure accurate inhibitory concentrations could be determined. In an attempt to estimate PPQ IC_50_ and IC_90_ of reduced susceptible isolates from 2013 tested on standard PPQ dilution range (0.9 to 674 nM), reanalysis was done to fit ‘zero-growth’ OD values at the extrapolated PPQ concentration of 53,905 nM to better estimate accurate dose–response curves [24].

### Ring-stage survival assay (RSA) for ART susceptibility

A portion of Cambodian and northeast Thai isolates was tested for sensitivity to ARTs using a ring-stage survival assay (RSA). Cambodian samples were tested for ex vivo RSA within 2–6 h of phlebotomy, without prior culture adaptation or parasite synchronization steps following published methods [25]. Briefly, samples with parasitaemias of ≤1 % were adjusted to 2 % haematocrit in culture media (0.5 % Albumax RPMI 1640 with 2.5 % AB serum), and cultured in a 48-well microplate with 700 nM DHA and 0.1 % DMSO in separate wells for growth control. Samples with >1 % parasitaemia were diluted to a parasitaemia range of 0.5–1 % by adding 50 % haematocrit human O+ red blood cells in 10 % serum-RPMI 1640 prior to adjusting to a 2 % haematocrit. The culture plate was then incubated for 6 h at 37 °C in a candle jar, after which culture medium was discarded. Cells were then washed, re-suspended in drug-free medium, and cultured for 66 h. Susceptibility to DHA was assessed microscopically on thin films by estimating the percentage of viable parasites, relative to control (% survival rate).

Similar methods were performed on Thai isolates, but instead of ex vivo testing on clinical samples, in vitro parasite culture was established for each sample and an in vitro RSA^0–3h^ was performed on 0–3-h post‐invasion rings obtained from culture‐adapted parasites following published methods [25]. Briefly, parasites cultures were synchronized utilizing 5 % d-sorbitol and 75 % Percoll to obtain 0 to 3-h post‐invasion rings which were adjusted to 0.5–1 % parasitaemia with a 2 % haematocrit in culture media. The following processes were performed as an ex vivo RSA, but parasites were cultured with mixed gas (5 % CO_2_, 5 % O_2_ and 90 % N_2_), instead of using a candle jar. As quality controls for the assay, the RSA^0–3h^ was also performed on *P*. *falciparum* reference clones W2, IPC-4884 and IPC-5202 (Malaria Research & Reference Reagent Resource, Manassas, Vermont, USA) to ensure an acceptable range of % survival rate was attained against these reference clones.

### Statistical analysis

Statistical analysis was performed using Graph-Pad Prism version 6.0 (GraphPad Software, Inc, San Diego, CA, USA). Parasite drug susceptibilities were expressed as median IC_50_s and IC_90_s for all isolates. Differences in susceptibility between groups were determined using non-parametric Mann–Whitney or Kruskal–Wallis tests and multiple comparison tests as appropriate. Correlations between PPQ ICs and % survival rate in the presence of DHA were analyzed by calculating Spearman’s correlation coefficient. In order to determine the PPQ susceptibility baseline, Grub’s test was used for outlier analysis of IC values attained from Cambodian isolates from 2010 to 2012 where no evidence of PPQ resistance was detected.

## Results

### HRP-2 dose response curves of *Plasmodium falciparum* isolates reveals severe ex vivo PPQ resistance

**Fig. 2:**
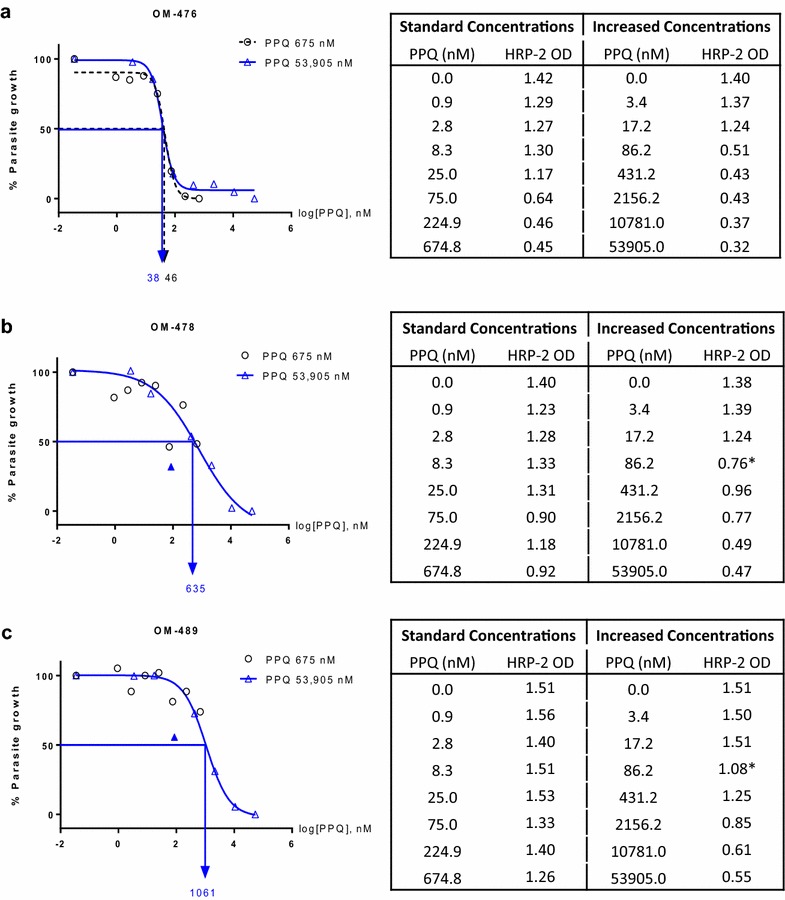
Representative dose response curves for *Plasmodium falciparum* isolates with severely reduced PPQ sensitivity. PPQ dose–response curves of *P. falciparum* isolates with a range of susceptibility. **a** is a PPQ-sensitive isolate (OM-476), while **b** and **c** are PPQ-resistant isolates (OM-478, OM-489). % parasite growth was plotted against log [PPQ concentration] using the standard PPQ concentration range from 0 to 674.8 nM (*black*) and an increased concentration range from 0 to 53,905 nM (*blue*), with IC_50_ for each indicated. *Filled symbols* represent outlier values excluded from curve fitting. Tables present HRP-2 OD values attained for individual isolates at each concentration range, while *asterisk* indicates values excluded prior to curve fitting

In 2013, evidence of severe ex vivo PPQ resistance not observed in prior years was detected in northern Cambodia when parasite isolates survived exposure at maximum PPQ levels (675 nM) normally employed in the ex vivo HRP-2 assay [24]. Since 2014, a serial dilution range to achieve higher maximum PPQ concentrations (3.4–53,905 nM) was used in addition to the standard dilutions to ensure accurate inhibitory concentrations could be determined. Figure 2 shows dose response curves for isolates with a range of susceptibilities based on standard PPQ dilutions (0.9–674.8 nM) compared to the higher concentration range (3.4–53,905 nM). For PPQ sensitive isolates, sigmoidal curves and reliable analysis were attained from both dilution ranges (Fig. 2a). However, reduced susceptibility isolates were able to grow in the maximum standard PPQ level (675 nM) preventing determination of accurate growth inhibition curves (Fig. 2b, c). Similar to a previous report [26], anomalous curves due to paradoxical parasite growth at high drug concentrations were observed in reduced susceptibility isolates. However, increasing maximum PPQ concentration to 53,905 nM conferred 100 % growth inhibition, attaining reliable dose–response curves. In an attempt to estimate PPQ IC_50_ and IC_90_ in these isolates, dose response curves were interpolated by excluding outliers to yield best-fit models (Fig. 2b, c).

### *Plasmodium falciparum* susceptibility to PPQ and MQ from 2010 to 2015

**Fig. 3:**
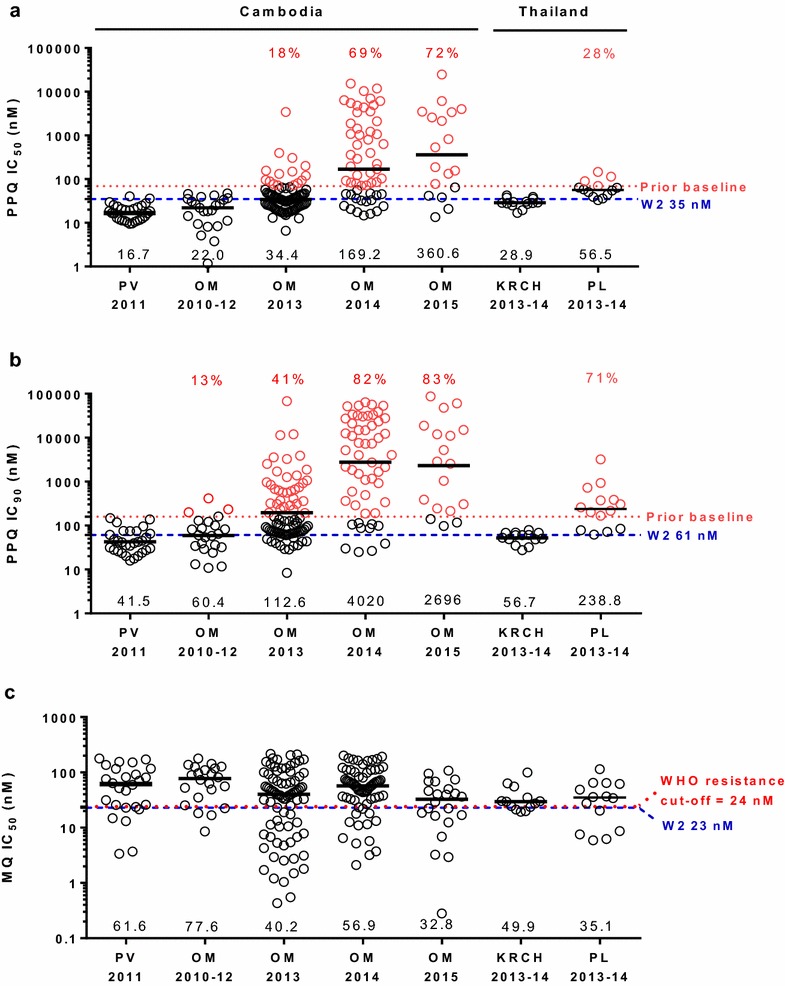
PPQ and MQ susceptibility of Cambodian and Thai isolates collected from 2010 to 2015.* Panels* **a** and **b**, respectively, present ex vivo *P. falciparum* IC_50_ and IC_90_ for PPQ, while MQ IC_50_ is shown in panel **c**. Site/year where samples were collected are indicated on the *X-axis*—Preah Vihear (PV), and Oddar MeanChey (OM) provinces of Cambodia, and Kwai River Christian Hospital (KRCH) in Kanchanaburi Province and Pursaron (PL) in Srisaket Province, Thailand, with median values of IC_50_ or IC_90_ denoted for each site/year with a *black bar* and indicated on *X-axis*. *Red dotted lines* indicate the PPQ susceptibility baseline of Cambodian isolates from 2010–2012 (**a**, **b**) and WHO values for suspected MQ resistance (**c**). The W2 susceptibility value is indicated by *blue dashed lines*. Isolates above prior year baseline for PPQ (IC_50_ > 69 nM, IC_90_ > 160 nM) are in *red*, with the proportion isolates above* baseline* indicated

Of 315 *P. falciparum* isolates tested using the ex vivo HRP-2 assay, IC results were evaluable in 222 isolates for PPQ and 230 for MQ. The in vitro assay was successful for all 14 isolates recovered among 27 cryopreserved samples. Figure 3a and b show PPQ IC_50_ and IC_90_ for *P. falciparum* isolates collected from Cambodia and Thailand from 2010 to 2015. Relative to a PPQ-sensitive W2 reference clone, Cambodian isolates from 2010 to 2012 were sensitive to PPQ with median IC_50_ and IC_90_ of 17–22 nM, and 41–60 nM, respectively. The emergence of PPQ resistance was detected in OM, Cambodia beginning in 2013, with parasite isolates surviving exposure to maximum tested PPQ levels (674 nM) in the ex vivo assay, with 18 and 40 % of isolates exceeding prior year baselines (IC_50_ > 69 nM and IC_90_ > 160 nM). More than half of isolates with elevated IC_90_ still had an IC_50_ below prior year baselines. PPQ resistance worsened in 2014–2015 with 70–80 % of isolates having IC_90_ and IC_50_ above prior baseline levels, in some cases by a thousand-fold over prior years.

In contrast, western Thai isolates from the same time period (2013–14) along the border with Myanmar were PPQ-sensitive with a median IC_90_ of 56.7 nM comparable to the 2010–12 Cambodian IC_90_ of 60.4 nM. Northeastern Thai isolates from 2013 to 14 collected from PL, roughly 100 km from Anlong Veng on the Thai–Cambodian border had intermediate PPQ sensitivity with a median IC_90_ of 238.8 nM. Kruskal–Wallis and multiple comparison analysis revealed significant elevations in median Cambodian isolate PPQ IC_50_ and IC_90_ values from 2013 to 2015, compared to prior years, as well as western Thai isolates from the same time period (*P* < 0.05). Modestly increased IC_50/90_ values for cryopreserved, northeastern Thai isolates were not statistically different from ex vivo isolates from western Thailand and Cambodia. The opposite trend was observed for MQ susceptibility (Fig. 3c) where Cambodian isolates developed increased susceptibility to MQ from 2013 to 2015, with lower median IC_50_ compared to prior years and to western Thai isolates (Kruskal–Wallis test, *P* = 0.008).

### Association of reduced PPQ susceptibility with clinical DHA-PPQ treatment failure

Emergence of PPQ resistance associated with DHA-PPQ treatment failure was identified in clinical studies conducted in Cambodia from 2010 to 13 [17]. *Plasmodium falciparum* isolates from those studies capable of ex vivo growth in the presence of 675 nM PPQ were more common in subjects suffering *P. falciparum* recrudescence within 42 days (43 %) than those without recrudescence (8 %; χ^2^ = 16.68; *P* < 0.001). Isolates from subjects with *P. falciparum* recrudescence had significantly increased IC_50_ and IC_90_ compared to those without recrudescence, most notably for IC_90_, which was three-fold higher than in those without recrudescence (median IC_50_ 38 vs 26 nM, *P* value of Mann–Whitney U test = 0.009; median IC_90_ of 201 vs 77 nM, *P* < 0.001). Elevated IC_50_ above prior baselines was more common in isolates from recrudescent cases (9/33 = 27 %), but was found in only 8 % of those without recrudescence (4/48; χ^2^ = 5.21; *P* = 0.023). Similar results were found for IC_90_ with values above prior years in 55 and 23 % of isolates from recrudescent and non-recrudescent cases, respectively (χ^2^ = 8.51; *P* = 0.004).

### Drug susceptibility profiles do not suggest cross-resistance between PPQ and ARTs

**Fig. 4:**
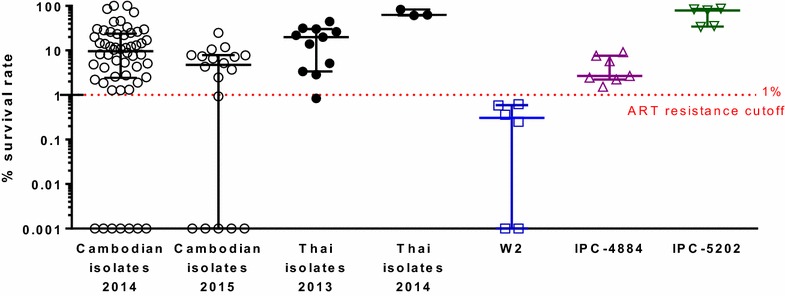
Ring-stage survival assay (RSA) for artemisinin susceptibility of *Plasmodium falciparum* isolates and reference clones. *Black unfilled* and *filled circles* represent  % survival rate in the ex vivo RSA for Cambodian isolates from Oddar MeanChey (OM) province and in vitro ^0−3h^ RSA for Thai isolates from Pursaron (PL) village in Srisaket province, respectively. *Blue squares* denote values of ART-sensitive (W2) clones while *triangles* indicate ART-resistant clones (IPC-4884-*purple* and IPC-5202-*green*) obtained from 5 to 7 independent in vitro ^0−3h^ RSA experiments. *Bars* represent median and interquartile range. Zero values of % survival rate were plotted as 0.001 % in logarithmic scale

To examine possible cross-resistance between PPQ and AS, 85 Cambodian isolates from 2014 to 2015 and 14 northeastern Thai isolates from 2013 to 2014 were tested in the RSA to measure % survival rate against DHA, and their association with PPQ IC_50_ and IC_90_ was assessed. Survival rates for 72 evaluable Cambodian isolates ranged from 0 to 100 % with a median of 7.7 and IQR of 2.0–16.4, while reference clone survival was as expected with medians of 0.3, 2.7, and 79.1 % for ART-sensitive W2, ART-resistant IPC-4884 and IPC-5202, respectively (Fig. 4). Based on an ART resistance cut-off above 1 % survival, only 22 % of Cambodian isolates (16/72) were sensitive to ARTs, while 78 % (56/72) were deemed resistant. No significant differences in the proportion of resistant isolates between 2014 (47/54 = 87 %) and 2015 (13/18 = 72 %) were found (χ^2^ = 2.13, *P* > 0.05), although there was a decrease in median survival rate for 2015 isolates (9.6), compared to those from 2014 (4.8), Mann–Whitney test, *P* = 0.017. Median survival rate for western Thai isolates was 24.0 with an IQR of 4.7–48.6, nearly all (13/14) were resistant to ARTs with survival rate above 1 %. Due to limited data available for Thailand, the change in ART resistance over time was not evaluated.

There was not a significant correlation between % survival rate in the presence of ARTs and PPQ IC_50_ or IC_90_ (Spearman ρ < 0.1, *P* > 0.05). ART-resistant Cambodian isolates (survival rate > 1 %) had higher PPQ ICs than sensitive isolates (survival rate ≤1 %), but differences were not statistically significant with IC_50_ of 448 nM for resistant isolates vs 268 nM for sensitive (*P* value for Mann–Whitney U test = 0.99), and IC_90_s of 9175 vs 4489 nM, respectively (*P* = 0.84).

## Discussion

Monitoring ex vivo drug susceptibility of *P. falciparum* revealed rapid progression of PPQ resistance in northern Cambodia between 2013 and 2015, following reports of severe clinically significant resistance in 2013 [15, 18], while western Thai isolates collected from the same time period remained sensitive to PPQ corresponding with a recent report of 94 % efficacy of DHA-PPQ in this region [5]. The northeastern Thai isolates showed modest worsening of PPQ resistance given their close proximity to the Cambodian parasite populations studied (within 100 km). Increased PPQ IC_50_ and IC_90_ associated with recrudescence in DHA-PPQ clinical studies from 2010 to 2013 corresponded with the first reports of DHA-PPQ clinical treatment failures in Cambodia [18, 19, 26]. The findings enforce those of recent studies, indicating the rapid expansion of PPQ-resistant parasites in Cambodia, as well as early signs that piperaquine resistance is emerging in Thailand. Given rapid emergence in Cambodia, and the fact that DHA-piperaquine has only recently been introduced on a large scale, the possibility for rapid worsening of resistance in Thailand is high. Limited treatment to choose from there is an urgent need for alternative therapies in the region. Whether the rapid expansion of PPQ resistance is due to increased transmission potential of PPQ-resistant isolates as observed for ART-resistant parasites [6] or slow-clearing parasites following DHA-PPQ treatment [27], requires further investigation.

The majority of both Cambodian and Thai isolates were resistant to ARTs in a RSA, consistent with a high prevalence of mutations in the propeller domain of the *P. falciparum kelch13* associated with ART resistance in nearly 100 % of isolates [15]. This supports emerging PPQ resistance on an ART resistance background given the high rate of clinical DHA-PPQ failures [18, 26], although there was no clear cross-resistance between PPQ and the ARTs, in part given the very high rate of ex vivo ART resistance. Similar to previous reports [15, 18, 26], the opposite trend was observed for MQ with increased ex vivo sensitivity noted in Cambodian isolates from 2013-2015. Studies also revealed the association of DHA-PPQ treatment failure with single copy of *P. falciparum* multi-drug resistance gene *(pfmdr1*) and decreased MQ IC_50_. The concomitant increase in MQ sensitivity coupled with worsening PPQ resistance could be explained by declining PPQ susceptibility within parasite population, being genetically distinct from those of MQ resistance. A recent population genetics study revealed emergence of PPQ resistance related to clonal expansion of MQ-sensitive parasites with a single *pfmdr1* copy (Parobek et al. pers. comm.)

Ex vivo parasite susceptibility to PPQ, particularly the IC_90_, appears to be a sensitive, field-expedient and relatively cost-effective marker to detect emerging PPQ resistance at sentinel sites in high-risk areas. Surveillance data first revealed the occurrence of PPQ-resistant parasites in Cambodia in 2010, a year before the clinical failure rate of DHA-PPQ began to increase. Although isolates from 2010 largely remained in the IC_50_ susceptible range, elevated IC_90_ was detected in three isolates, two of which were in patients who failed DHA-PPQ treatment [28]. In 2013 when clinical failure for DHA-PPQ was prevalent, IC_90_ was elevated in 40 % of isolates though more than half still had IC_50_ in the sensitive range. A rapid progression of resistance was detected in years following with rapid rises in both IC_90_ and IC_50_ for most isolates. Decreased parasite susceptibility to PPQ was associated with DHA-PPQ treatment failure, notably for IC_90_, which was three-fold higher in subjects failing treatment. IC_90_ appeared to better elucidate shifts in resistance patterns over time and their association with treatment outcomes compared to IC_50_. Anomalous curves observed with PPQ-resistant isolates in the HRP-2 assay required PPQ concentrations up to 53,905 nM (>25 times higher than the maximum concentration used by Duru et al. [26] before 100 % growth inhibition producing an interpretable, sigmoidal, dose–response curve could be achieved (Fig. 2). Similar to findings with HRP-2, a recent study reported poor performance of the standard hypoxanthine uptake assay due to the high frequency of non-interpretable curves observed with PPQ-resistant isolates. The anomalous curves produced suggested a paradoxical increase in parasite growth at PPQ concentrations above 100–200 nM, reflecting a resistance mechanism induced at physiological concentrations (~200 nM) observed in the blood of patients treated with DHA-PPQ [26, 29]. Evidence of paradoxical growth at high concentrations of drugs was previously described in non-ART drug assays [30]. This was suggested to reflect biological properties of drugs, including mechanism of action. Amendment of the lower constraint of the sigmoid model was suggested to provide a more accurate measurement. However, this is not the case for the paradoxical growth seen among resistant isolates in the PPQ assay demonstrated here, or in another recent report [26], and may represent a phenomenon of PPQ-resistant isolates.

To compensate for these challenges for interpretation, a novel in vitro assay for PPQ resistance, the PPQ survival assay (PSA), was designed to mimic in vivo exposure of parasites to a pharmacologically relevant dose of PPQ (200 nM) for 48 h with the  % parasite survival rate measured at 24 h after drug exposure [18, 21, 26]. A 48-h drug incubation period allows adequate PPQ exposure for all parasite stages from 0-h rings to 48-h schizonts, given PPQs having long clinical half-life (~9 days) [26, 29]. As a result, the assay does not assess parasite susceptibility to PPQ at a specific parasite stage, but rather the entire life cycle. PSA was demonstrated to be a useful tool to differentiate PPQ resistance among individual fresh isolates, and the % survival rate was found to correlate with DHA-PPQ treatment outcome. Future investigations employing this method are likely to further elucidate emerging piperaquine resistance, although the requirement for multiple individual microscopy readings on each sample limits assay throughput to some degree. The choice of method (PSA, HRP-2, etc.) should be based on available resources and experience. In our view, consistency of application, reproducibility and interpretability of results across time and place are far more important than the technique selected.

The limited sample size for Thai isolates, as well as lack of more recently collected samples are important limitations of the current study. This was mitigated by the sequential approach employed at the sentinel sites, increasing the degree of confidence that median values presented here were reflective of relative drug sensitivities at the community level. Isolates from the Thai–Myanmar border remained highly sensitive to PPQ despite ART resistance comparable to the levels seen in Cambodia, as well as modest MQ resistance. Samples were collected before the widespread use of PPQ in Thailand when AS-MQ remained the first-line drug. While there has clearly been rapid expansion of resistance in northern Cambodia, there appears to be only limited spread across the Thai border, with isolates from nearby PL, Thailand having only moderate declines in PPQ susceptibility. This may be due in part to a lower burden of disease in northeastern Thailand permitting more stringent control measures, and anti-malarial drug administration limited to well-resourced government public health clinics. Regardless, ongoing monitoring is imperative in this region.

Several reports have suggested that ART resistance could promote development of resistance to PPQ, as ART-resistant isolates surviving in the presence of ACT are more likely to spontaneously develop resistance to partner drugs [18, 21]. The association between PPQ resistance and the K13 mutation likely reflects selection of PPQ-resistant parasites as those initially resistant to ARTs remain viable well after ACT treatment has been completed. This has been supported by apparent correlations between increasing DHA-PPQ failure rates on a background of ART-resistant isolates with K13 mutations [17–19]. A recent study reported that all PPQ-resistant isolates carried the K13 mutation, although direct correlation between PPQ and ART susceptibility was not clear as PPQ-sensitive isolates also carried K13 mutation [26]. This may be due to the high prevalence of K13 in the parasite populations studied, and limits potential analysis within the present dataset. Cutoff IC_50_ or IC_90_ values for PPQ resistance have yet to be defined, we have not made an effort here to compare % survival rate to ART between PPQ ‘resistant’ and ‘sensitive’ isolates. We performed a Spearman correlation analysis between % survival rate and PPQ IC_50_ or IC_90_ and also compared PPQ IC_50_/IC_90_ between ART-sensitive (RSA ≤ 1 %) and resistant isolates (RSA > 1 %). A significant correlation between ART and PPQ resistance was not found (see Additional file 1).

Making use of these data for public health purposes presents important challenges, although there does appear to be a key opportunity in the inverse resistance pattern between PPQ and MQ. PPQ resistance appears to be developing in MQ-sensitive parasites with single-copy *Pfmdr*-*1* and low MQ IC_50_, as PPQ-resistant isolates in a recent study (PSA survival rates ≥10 %) all had a single *Pfmdr*-*1* copy and were largely MQ sensitive [26]. Correspondingly, a parasite population genetics study identified the development of resistance to PPQ and MQ in genetically distinct parasite populations (Parobek et al. pers. comm.). In the interim, the national control programme in Cambodia has reverted to the re-introduction of AS-MQ ACT in selected areas of PPQ resistance, and previous studies have observed excellent cure rates using AS-MQ as rescue therapy for DHA-PPQ treatment failures [17, 28]. Recent investigations have proposed triple-drug therapy such as simultaneous AS, MQ and PPQ [18], although the safety of such combinations has not been studied. Further, the possibility of inducing simultaneous resistance to all three drugs amidst rising resistance to the individual components would argue for preserving the advantages afforded by inverse MQ and PPQ resistance patterns by rotating two drug ACT combinations sequentially, combined with inpatient follow-up of all cases to ensure compliance [21]. Doing so in a carefully coordinated fashion on a biannual basis would permit adequate time to restore at least partial partner drug sensitivity, while holding the current second-line therapy in reserve for clinical treatment failures. Atovaquone-proguanil used as part of public health containment activities in Cambodia may prove to be a useful albeit costly third-line agent, as isolates from the same areas of PPQ resistance in northern Cambodia reported here were recently shown to remain highly susceptible to atovaquone (ATQ) in vitro without evidence of pre-treatment mutations in the cytochrome b gene codon 268 marker for ATQ resistance [24]. However, this liability is well known, with several studies reporting rapid development of single point mutations in cytochrome b conferring ATQ resistance during treatment [31–33]. Neither approach will adequately address the current high-grade ART resistance in the region, although there is hope they may extend the life of current ACT until novel compounds in development, such as the spiroindolones [34] and synthetic ozonides [35], become available.

## Conclusions

The study revealed the rapid expansion of PPQ-resistant parasites in northern Cambodia during 2013–2015, while Thai isolates remained sensitive to PPQ, or showed modest worsening of resistance for those in close proximity to the Cambodian study sites. The opposite trend was observed for MQ with increased ex vivo sensitivity noted in Cambodia. Rapid progression of PPQ resistance associated with the treatment failures of the latest ACT regimen, DHA-PPQ, in northern Cambodia. The findings enforce those of recent studies, indicating the rapid expansion of PPQ-resistant parasites in Cambodia, as well as early signs that piperaquine resistance is emerging in Thailand. Limited drugs of choice in this region highlights the demand alternative therapies. While the re-introduction of AS-MQ ACT remained a treatment choice for PPQ-resistance areas at time of writing, careful monitoring for re-emergence of MQ resistance and/or simultaneous resistance to all three drugs (AS, MQ and PPQ), as well as of the spread of PPQ resistance to nearby countries, remain crucial to address the resistance crisis.

## Additional file

**Additional file 1.** Artemisinin and piperaquine susceptibility profiles do not suggest cross resistance.

## Abbreviations

AS: artesunate
MQ: mefloquine
PPQ: piperaquine
IEV: immediate ex vivo
HRP-2: histidine-rich protein 2
ELISA: enzyme-linked immunosorbent assay
IC_50_: 50 % inhibitory concentration
IC_90_: 90 % inhibitory concentration
RSA: ring-stage survival assay
PSA: piperaquine survival assay
ACT: artemisinin combination therapy
PCR: polymerase chain reaction
TACT: triple artemisinin combination therapy
PV: Preah Vihear
OM: Oddar Meanchey
PL: pursaron
KRCH: Kwai River Christian Hospital
OD: optical density
IQR: interquartile range
ART: artemisinin
*Pfmdr1*: *P. falciparum* multi-drug resistance gene
ATQ: atovaquone
WHO: World Health Organization
