# Supplementary material for: Ex vivo piperaquine resistance developed rapidly in Plasmodium falciparum isolates in northern Cambodia compared to Thailand
Source: Malar J. 2016 Oct 21;15:519. doi: 10.1186/s12936-016-1569-y (PMC5075182; doi:10.1186/s12936-016-1569-y)

Additional file 1

Artemisinin and piperaquine susceptibility profiles do not suggest cross resistance.

PPQ IC_{50} and IC_{90} of arteminsisnin-sensitive (%survival rate ≤ 1) and resistant isolates (%survival rate > 1) were compared; median values of each group and *P*-values for the Mann-Whitney U test are indicated. There were no difference in PPQ IC_{50} and IC_{90} between arteminsisnin-sensitive and resistant isolates.

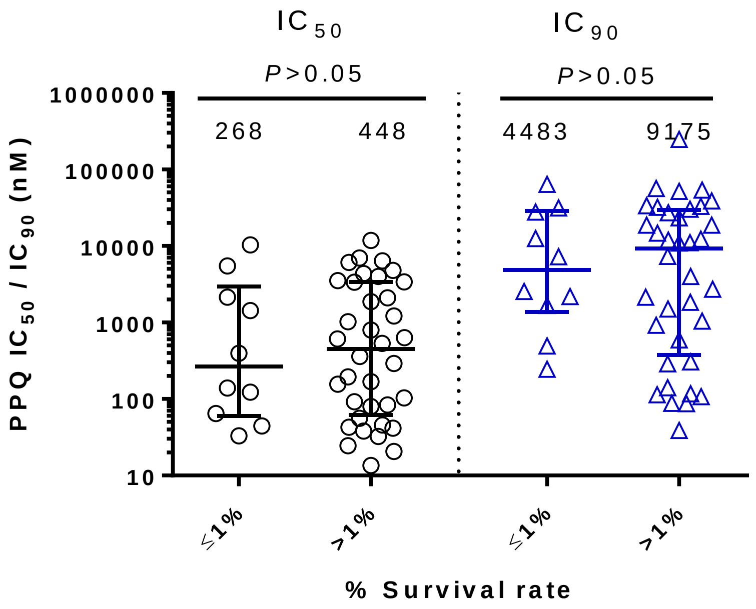

Supplement: Supplementary file 1 — Additional file 1. Artemisinin and piperaquine susceptibility profiles do not suggest cross resistance. [file 12936_2016_1569_MOESM1_ESM.pdf]
